# Snail Contributes to the Maintenance of Stem Cell-Like Phenotype Cells in Human Pancreatic Cancer

**DOI:** 10.1371/journal.pone.0087409

**Published:** 2014-01-29

**Authors:** Wei Zhou, Ran Lv, Weilin Qi, Di Wu, Yunyun Xu, Wei Liu, Yiping Mou, Liewei Wang

**Affiliations:** 1 Department of General Surgery, Sir Run Run Shaw Hospital, School of Medicine, Zhejiang University, Hangzhou, P. R. China; 2 Departments of Anesthesiology, Sir Run Run Shaw Hospital, School of Medicine, Zhejiang University, Hangzhou, P. R. China; 3 Department of Molecular Pharmacology and Experimental Therapeutics, Mayo Clinic, Rochester, Minnesota, United States of America; University of Colorado, Boulder, United States of America

## Abstract

Snail, a potent repressor of E-cadherin expression, plays a key role in epithelial-to-mesenchymal transition (EMT) in epithelial cancer. Recently, EMT and stemness programs are found linked together. In the current study, the expression of Snail and its contribution to cancer stem cell (CSC) marker expression, invasiveness, self-renewal, clonogenicity, and tumorigenicity of pancreatic cancer cells were studied. Our results showed that Snail was highly expressed in CSC^high^ cell line Panc-1. Stable, short hairpin RNA (shRNA)-mediated Snail knockdown decreased invasion in Panc-1 cells, in line with increased E-cadherin expression and its translocation from the nucleus to the membrane. Snail silencing in Panc-1 also inhibited CSC marker ALDH expression, together with decreased sphere and colony forming capacity, which was highly consistent with the expression of stem cell associated transcription factors like Sox2 and Oct4. In mouse xenograft models, knockdown of Snail led to a reduced number of tumor-bearing mice and a reduced average size of tumors, which had a stronger membrane staining of E-cadherin and lighter staining of Oct4. Collectively, these findings implicate Snail is required for the maintenance of stem cell-like phenotype in pancreatic cancer, and inhibition of Snail could be an efficient strategy to treat pancreatic cancer by targeting CSCs.

## Introduction

Pancreatic ductal adenocarcinoma is a highly aggressive epithelial cancer with a reported 5-year survival rate of approximately 5%[1]. Only 20% of pancreatic cancer patients are eligible for surgical resection, and metastatic disease frequently develops even after surgery, while current chemo- and radio-therapies are largely ineffective[2]. Therefore, Understanding the molecular events underlying the development and progression of pancreatic cancer is urgently needed, which may hold the key to the development of more efficacious and novel therapeutic strategies.

An increasing amount of scientific evidence indicates that tumors contain a small subpopulation of cells, i.e., cancer stem-like cells (CSCs) or cancer-initiating cells (CICs), which exhibit a self-renewing capacity, resistant to conventional chemotherapy and are responsible for therapy failure, cancer relapse and metastasis [3]. Although the CSCs hypothesis suggests that tumors can arise from stem or progenitor cells, studies from some laboratories indicate that epithelial-mesenchymal transition (EMT), a developmental process in which cells lose epithelial characteristics and acquire mesenchymal properties such as increased motility and invasion, can endow cells with stem-cell like characteristics[4]–[6].

EMT is induced by repression of E-cadherin expression by EMT regulators such as Snail, Slug, and Twist. The Snail family of zinc-finger transcriptional repressors directly represses E-cadherin in vitro and in vivo via an interaction between their COOH-terminal region and the 5′-CACCTG-3′ sequence in the E-cadherin promoter [7]. In human colorectal cancer cells, overexpression of Snail was reported to induce not only EMT but also a CSC-like phenotype, which enhanced cell migration and invasion in vitro and an increase in metastasis formation in vivo[8]. Studies have also shown that Snail plays an essential role in the progression and metastatic process of human pancreatic cancer[9], [10]. In clinical setting, Snail overexpression has previously been associated with poorer prognosis and a more invasive phenotype in many malignancies[11]–[13]. However, few reports exist regarding the link between Snail expression and the gain of pancreatic cancer stem cell properties. We therefore evaluated the Snail's function on stem cell marker expression, self-renewal capacity in pancreatic cancer cell line in vitro and xenograft tumors formation in vivo. Our work reveals that gene regulation mediated by Snail may support human pancreatic cancer growth by maintaining the pancreatic cancer stem cell compartment.

## Materials and Methods

### Cell culture

The human pancreatic cancer cell lines Panc-1 and BxPC-3 were obtained from the American Type Culture Collection (Manassas, VA). Cells were cultured and maintained in DMEM medium supplemented with 10% fetal bovine serum (Gibco/Invitrogen, CA), penicillin-streptomycin (Flow Laboratories, Rockville, MD). Both cell lines were maintained in a humidified atmosphere at 37°C with 5% CO2. Gross cell morphology for the presence or absence of morphologic characteristics consistent with EMT was assessed by two observers blinded to the treatment conditions. Images of cell lines were taken using a Nikon Eclipse TS100 inverted microscope and Pro-MicroScan camera (Oplenic).

### Evaluation of aldehyde dehydrogenase activity

Aldefluor substrate (2.5 µl, Aldagen, Inc., Durham, NC) was added to 1×10^6^ tumor cells in 500 µl assay buffer and incubated for 60 min at 37°C. Cells were analyzed on a FACSCalibur flow cytometer (Becton Dickinson) according to the instructions of the manufacturer. Treatment of cells with 5 µl of the ALDH inhibitor diethylamino-benzaldehyde (DEAB) served as negative control. Lentiviral vectors without fluorescence were used for cell transfection during FACS Analysis.

### Sphere formation assay

Sphere formation assay was performed as described elsewhere[14]. In brief, cells were plated in six-well ultralow attachment plates (Corning Inc., Corning, NY) at a density of 1,000 cells/ml in DMEM supplemented with 1% N2 supplement (Gibco, Grand land, NY), 2% B27 supplement (Gibco, Grand island, NY), 20 ng/ml human platelet growth factor (Sigma-Aldrich), 100 ng/ml epidermal growth factor (PeproTech, Rocky Hill, NJ) and 1% antibiotic-antimycotic (Invitrogen) at 37°C in a humidified atmosphere of 95% air and 5% CO2. Sphere cultures were passaged after 7∼10 days. To passage spheres, media was removed and spheres were collected and incubated at room temperature for 5 min in 0.05% trypsin (Solarbio, Beijing, China) and observed under the microscope to verify dissociation. The cells obtained from dissociation were sieved through a 40-µm filter and counted by counter using trypan blue dye before replating.

### Soft agar assay

To assess clonogenic potential, the colony formation assay was performed as follows. Each well of a six-well culture dish was coated with 2 ml bottom agar mixture (DMEM, 10% (v/v) FCS, 0.6% (w/v) agar). After the bottom layer solidified, 2 ml top agar medium mixture (DMEM, 10% (v/v) FCS, 0.3% (w/v) agar) containing 1×10^4^ cells was added, and the dishes were incubated at 37°C for 3 weeks. Plates were stained with 0.5 ml of 0.005% crystal violet for 1 h and then a dissecting microscope was used to count the number of colonies >50 cells [15].

### Transwell invasion assay

For invasion assay, the 24-well plate Transwell system with an 8-µm pore size polycarbonate filter membrane (Corning Costar, Corning, NY) was used. 1×10^5^ cells in 100 µl serum-free medium were added to the top chamber coated with Matrigel (BD Bioscience, Bedford, MA). The lower chamber contained 10% FBS containing medium. The cells were incubated for 48 h and cells that had invaded through the Matrigel-coated membrane were fixed and stained with crystal violet, then counted under a light microscope in four random fields in a blinded fashion.

### Real-time RT-PCR analysis for gene expression

For real-time RT-PCR analysis, the total RNA of cells was extracted by using the Trizol kit (Invitrogen, Carlsbad, CA). cDNA was synthesized using equivalent amounts of total RNA (1 µg) with random primers in a 20 µl reverse transcriptase reaction mixture (Promega, Madison, WI). Real-time PCR primers were designed and purchased from Ruisai Inc (Shanghai, China) as follows:

Snail, forward (5′-GCTGCAGGACTCTAATCCAGA-3′)

and reverse (5′-ATCTCCGGAGGTGGGATG-3′);

Slug, forward (5′-AGCGAACTGGACACACATAC-3′)

and reverse (5′-TCTAGACTGGGCATCGCAG-3′);

Twist 1, forward (5′-CACTGAAAGGAAAGGCATCA-3′)

and reverse (5′-GGCCAGTTTGATCCCAGTAT-3′);

ZEB1, forward (5′-CGAGTCAGATGCAGAAAATGAGCAA-3′)

and reverse (5′-ACCCAGACTGCGTCACATGTCTT-3′);

ZEB2, forward (5′-GAGTTGATGCCTCGGCTATTGC-3′)

and reverse (5′- CTGGACATTGAGCTGCTTCGATC-3′);

GAPDH, forward (5′-GGTGTGAACCATGAGAAGTATG-3′)

and reverse (5′- GATGGCATGGACTGTGGTCAT-3′).

The amplification was carried out in a total volume of 20 ul containing 1 ul of each primer, 10 ul LightCycler FastStart DNA Master SYBR green I (Roche Diagnostics, Pleasanton, CA) and 1 ul of 1∶10 diluted cDNA. PCR reactions were prepared in duplicate and heated to 95°C for 2 min followed by 40 cycles of denaturation at 95°C for 15 sec, annealing at 55°C for 20 sec, and extension at 72°C for 20 sec. All assays were performed in triplicate and were calculated on the basis of ^ΔΔ^Ct method. The n-fold change in mRNAs expression was determined according to the method of 2–^ΔΔ^CT.

### Lentiviral-mediated RNAi of Snail

The pcDNA6.2-GW/EmGFP-miR vector was purchased from Invitrogen (Carlsbad, CA). Double-stranded shRNAs targeting human Snail were designed by BLOCK-i T™ RNAi Designer. The targeted sequence 1 was 5′-GCCTAACTACAGCGAGCTG-3′; targeted sequence 2 was 5′-GGATCTCCAGGCTCGAAAG-3′. Both targeted sequences were verified as specific for Snail by Blast searching against the human genome. Universal non-targeting control shRNA (sequences: 5′-TGCTGAAATGTACTGCGCGTGGAGACGTTTTGGCCACTGACTGACGTCTCCACGCAGTACATTT-3′; 5′-CCTGAAATGTACTGCGTGGAGACGTCAGTCAGTGGCCAAAACGTCTCCACGCGCAGTACATTTC-3′) was used as negative control. shRNAs were synthesized and cloned into pcDNA6.2-GW/EmGFP-miR, then transferred into the lentiviral expression plasmid pLenti6/V5-DEST with the Gateway recombination technology. Lentiviral production was done by transfection of 293 T cells with shRNA or negative control plasmid and packaging Mix (Invitrogen) in the presence of POLOdeliverer™ 3000 Transfection Reagent (Ruisai Inc, shanghai, China). Supernatants were collected 48 h after transfection and then were filtered; the viral titers were then determined by real-time PCR. Subconfluent cells were infected with lentivirus at a multiplicity of infection of 50 in the presence of 8 µg/ml polybrene (Sigma-Aldrich). Panc-1 cells with stable silencing of Snail gene were selected with 2 ug/ml of Blasticidin for 4 weeks. Then cells were cultured with 1 ug/ml Blasticidin. Another negative control shRNA plasmid (sc-108060) from Santa Cruz Biotechnology, which encodes a scrambled shRNA sequence, was used for transient transfection according to the protocol.

### Western blot analysis

For whole cell protein extraction, cells were lysed with ice-cold RIPA buffer containing 1 mM PMSF and centrifuged at 14,000 g for 5 min. Supernatant containing the isolated protein was quantified by a commercially available modified Bradford assay (Bio-Rad Laboratories, Hercules, CA). Western blot protein samples were prepared by boiling isolated proteins with denaturing sample buffer. Equal amounts of protein were resolved by SDS-PAGE and transferred to nitrocellulose membranes. The membranes were then blocked with 5% nonfat dry milk in TBS and 0.1% Tween 20 for 1 h and probed with the appropriate primary antibody overnight at 4°C. The membranes were then washed and incubated with the appropriate horseradish peroxidase–conjugated secondary antibody (Sigma Aldrich, St Louis, MO) for 1 h at room temperature. Membranes were then washed and protein bands were visualized by using a commercially available enhanced chemiluminescence (ECL) kit (Thermo Scientific). To verify the accuracy of loading of protein isolated from whole-cell lysate, the blots were stripped, washed and reprobed with GAPDH antibody (Bioworld) as a loading control. Images were visualized using the ECL Detection System (Amersham, Arlington Heights, IL). Antibodies used for Western blot analyses were as follows: rabbit mAb anti-Snail, rabbit mAb anti-E-Cadherin, rabbit mAb anti-vimentin, rabbit mAb anti-Bmi1, rabbit mAb anti-Nanog, rabbit mAb anti-Oct4, rabbit mAb anti-Sox2 (Cell Signaling Technology, Inc.). Densitometry of Western blots was analyzed by using Image J software.

### Immunofluorescence microscopy

Cells were grown on glass coverslips, fixed in 4% formaldehyde in PBS for 10 min, permeabilized with 0.2% Triton X-100 for 10 min, and blocked in 10% goat serum in PBS-0.2% Tween for 1 h. Coverslips were incubated with E-cadherin antibody (BD Biosciences, 1∶200 dilution) in blocking solution for 1 h at room temperature. After washing the cells with PBS-0.2% Tween, we incubated the cells for 1 h with goat anti-rabbit IgG Alexa 594 (1∶1000 dilution; Invitrogen). Nuclei were counterstained with DAPI. The slides were washed extensively with PBS and mounted with Fluoromount-G. Samples were photographed using immunofluorescence microscope.

### In vivo analysis of tumor growth

All procedures involving animals were approved by the Animal Care and Use Committee of Zhejiang University School of Medicine (ZYXK2010-0149). Single cells suspensions (2×10^5^ in a total volume of 100 µL of 1/1 (v/v) PBS/Matrigel) were injected subcutaneously into the right and left midabdominal area of male nude mice (BALB/c strain) aged 8 weeks. The tumor size monitored daily with calipers and the tumor volume was calculated according to the formula (Length×Width^2^)/2. Animals underwent autopsy at 28 days after cell implantation and tumor growth was assessed.

### Immunohistochemistry analysis of xenograft tissue

The subcutaneous tumors formed in mice were fixed in 10% phosphate-buffered formalin and embedded in paraffin. 4-µm thick sections were deparaffinized using xylene, and hydrated by a graded series of ethanol washes. Endogenous peroxidase activity was quenched by 10-min incubation in 3% H_2_O_2_. After incubation with blocking solution for 30 min, sections were incubated with primary antibody from BD Biosciences (anti-Snail, 1∶200 dilution; anti-E-cadherin, 1∶200 dilution, and anti-Oct4, 1∶300 dilution)for 1 h, a biotinylated secondary antibody for 20 min and then with streptavidin horseradish peroxidase (HRP) for 10 min. The antibody was visualized with diaminobenzidine (DAB) chromogen, and sections were counterstained with H&E.

### Statistical analysis

The experiments were repeated at least two times. Results are expressed as mean±SD. Statistically significant differences were determined by Student's t test for independent samples when appropriate using SPSS statistical analysis software (version 13.0) (SPSS, Inc., Chicago, IL). Significant differences among groups were calculated at P<0.05.

## Results

### Snail expression and stem cell marker ALDH in pancreatic cell lines

**Figure 1:**
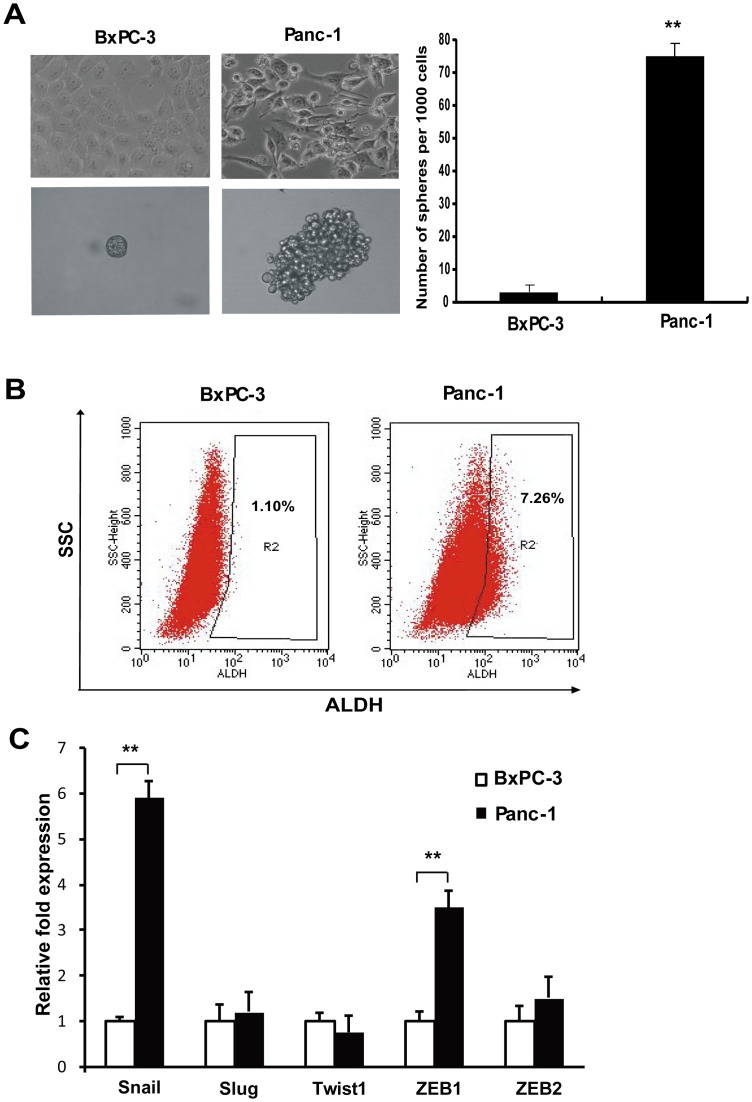
Differences of epithelial-mesenchymal features and CSC properties in Panc-1 and BxPC-3 cells. A. Morphology of Panc-1 and BxPC-3 cells and their spheres. Note that Panc-1 cells have more spindle-shaped mesenchymal populations and can form more and larger spheres. ** P<0.01 compared with BxPC-3. B. ALDH activity in Panc-1 and BxPC-3 cells. Dot plots of cells analyzed by flow cytometry for ALDH activity. Cells were treated with Aldefluor substrate in the presence or absence of ALDH inhibitor DEAB. After treatment, the samples were analyzed by flow cytometry for the presence of ALDH^high^ cells. The values presented are the averages of three independent experiments. C. Real-time RT-PCR quantifing Snail, Slug, Twist1, ZEB1, and ZEB2 mRNA expression in Panc-1 and BxPC-3 cells. Bar graphs show the ratio of the expression level in Panc-1 cells to that in BxPC-3 cells. ** P<0.01.

Under the microscope, BxPC-3 cells were morphologically epithelial in nature. In contrast, Panc-1 cells were mixed populations of epithelial and spindle-shaped mesenchymal type cells (Figure 1 A). Using flow cytometry, poorly-differentiated cell line Panc-1 was characterized as CSC^high^ cell with more ALDH^high^ population, while well-differentiated cell line BxPC-3 as CSC^low^ cell with less ALDH^high^ population (Figure 1 B). Sphere formation assay also revealed that Panc-1 formed more and larger spheres than BxPC-3 did (Figure 1 A). To gain insight into the crucial role of the EMT regulators, we examined the basal expression of Snail, Slug, Twist1, ZEB1 and ZEB2 in Panc-1 and BxPC-3. Notably, real-time RT-PCR analyses showed that Panc-1 cells had a significantly higher expression of Snail and ZEB1 mRNA (approximately 6-fold and 4-fold respectively, P<0.01) in comparison to BxPC-3 cells (Figure 1 C). There was a correlation between poor differentiation, Snail and ZEB1 expression levels and sphere-forming capacity in these two pancreatic cancer cell lines. We chose the Panc-1 cell line for later Snail silencing experiments due to its relatively high expression level of Snail and excellent lentiviral transfection efficiency.

### Snail induces EMT-like changes in pancreatic cancer cell

To examine the role of Snail in EMT induction and CSC generation, we produced a stably Snail knockdown Panc-1 cell line (Panc-1/shSnail) by lentiviral transfection.

**Figure 2:**
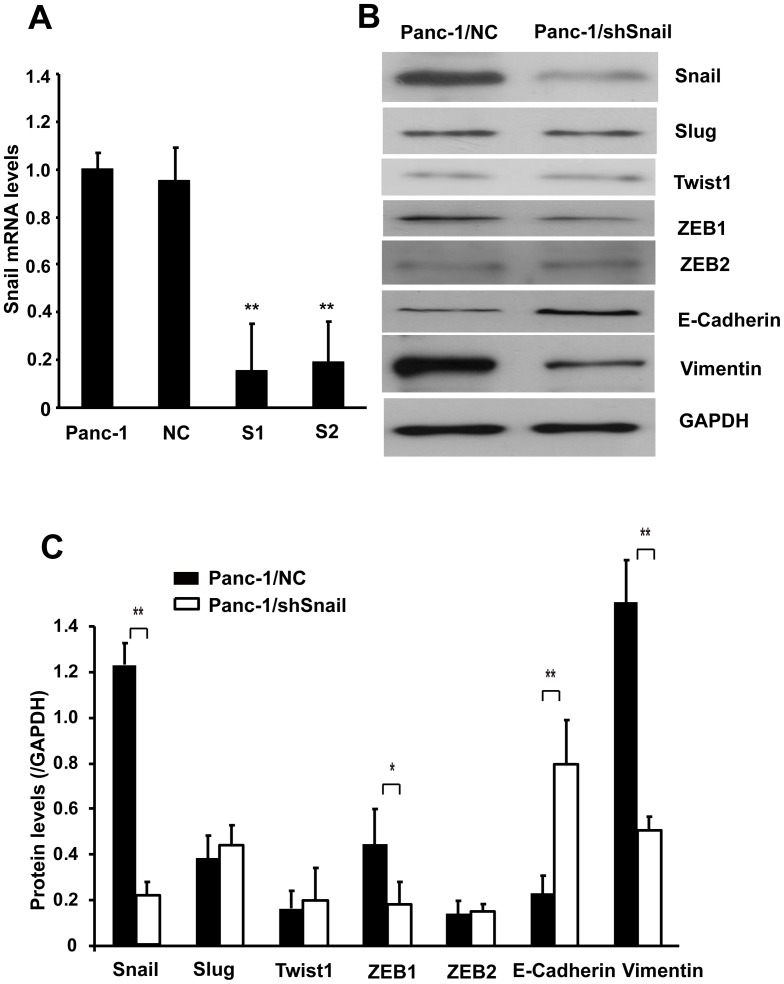
Changes of epithelial-mesenchymal makers after Snail silencing in Panc-1 cells. A. Snail mRNA expression of stable shSnail-expressing (S1, S2) and negative control shRNA-expressing (NC) Panc-1 clones. Values are the averages and standard deviations of triplicate measurements. **P<0.01 compared with NC. B. Western blot showing epithelial-mesenchymal markers Snail, Slug, Twist1, ZEB1, ZEB2, E-cadherin and vimentin in Panc-1 cells transfected with lentivirus-mediated negative control shRNA (Panc-1/NC) or shSnail (Panc-1/shSnail). GAPDH was used as loading control. C. Quantification of protein levels of Snail, Slug, Twist1, ZEB1, ZEB2, E-cadherin and vimentin in Panc-1/NC and Panc-1/shSnail cells. * P<0.05, **P<0.01.

Two independent stable shSnail-expressing clones (S1, S2) and a stable negative control shRNA clone (NC) were analyzed for Snail mRNA expression. S1 and S2 showed up to 80% downregulation of Snail mRNA levels, whereas control clone did not exhibit any significant reduction in Snail mRNA (Figure 2A). We chose S1 clone as Panc-1/shSnail for following experiment because of its higher extent of Snail silencing. Knockdown of Snail in Panc-1 cells was confirmed by Western blot analysis (Figure 2B and C). Rabbit mAb anti-Snail was validated against cell lysates from Panc-1, MIA-PaCa2, BxPC-3, and Capan-1 cell lines. The staining pattern was similar to that of previously published data (Figure S1) [9], [16]. To rule out heterogeneity caused by stable transfection and selection protocol, we used another negative control shRNA plasmid (sc-108060) for transient transfection. Real-time RT-PCR and Western blot revealed no significant difference of Snail expression between stably and transiently transfected control clones (Figure S2).

**Figure 3:**
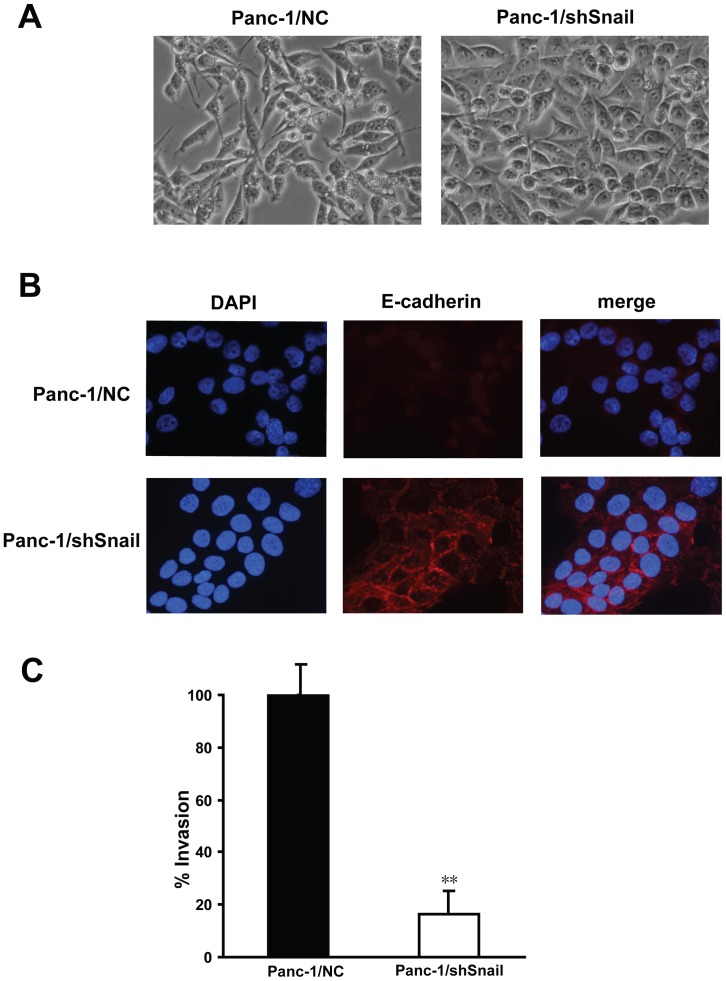
Changes of epithelial-mesenchymal features after Snail silencing in Panc-1 cells. A. Morphological alterations of Panc-1 cells after Snail silencing indicate a change in the cellular growth pattern from a mesenchymal towards an epithelial phenotype. B. Immunofluorescence staining for the expression and cellular localization of E-cadherin in stable clones of Panc-1/NC and Panc-1/shSnail. Nuclear DNA was detected by DAPI staining. Stable Snail knockdown leads to an increased expression of E-cadherin and its translocation from the nucleus to the membrane. C. Snail silencing inhibits Panc-1 invasiveness in in vitro Matrigel invasion assays. **P<0.01 compared with Panc-1/NC. Data shown here are the mean ± SD of three experiments.

After lentivirus infection, blinded investigators observed differences in the gross appearance of cells. Stable infection of Panc-1 cells by shSnail significantly increased intercellular adhesion and cobble-stone-like shape, as compared with control cells (Figure 3A). Next, we evaluated the expression and localization of E-cadherin using immunofluorescence staining. Compared with Panc-1/NC cells, Panc-1/shSnail cells had increased levels of expression of E-cadherin and relocation of E-cadherin from the nuclear compartment to cell plasma membrane (Figure 3B). These changes were typical of cells with an epithelial phenotype, indicating that the cells were undergoing mesenchymal-to-epithelial transition (MET) after Snail silencing. To further confirm the observation, we assessed the expression of epithelial adhesion molecule E-cadherin, mesenchymal cell marker vimentin and other EMT transcription factors Slug, Twist1, ZEB1 and ZEB2 by Western blotting on cell lysates. As expected, after silencing of Snail in Panc-1, expression of the E-cadherin was observed to increase. Conversely, a decrease in the expression of vimentin and ZEB1 was observed after Snail knockdown. No significant differences were observed in protein levels of Slug, Twist1 and ZEB2 (Figure 2 B, C). These results show a clear relationship between Snail and E-cadherin, and also suggest a direct or indirect interaction between Snail and ZEB1, both of which hold the potential to repress E-cadherin transcription.

### Snail increases cell invasion ability in pancreatic cancer cell

Since the processes of EMT have been linked with cell invasion, we next asked if Snail expression has some effect on cell invasion capacity in pancreatic cancer cells. Using the Matrigel in vitro invasion assay, we found Panc-1 cells with Snail silencing had significantly decreased capacity for invasion when compared to control cells (Figure 3 C). These data confirm that Snail expression enhances the invasive capacity of pancreatic cancer cells, and the inactivation of Snail leads to MET with less invasive characteristics.

### Snail enhances stem-cell like properties in pancreatic cancer cells

**Figure 4:**
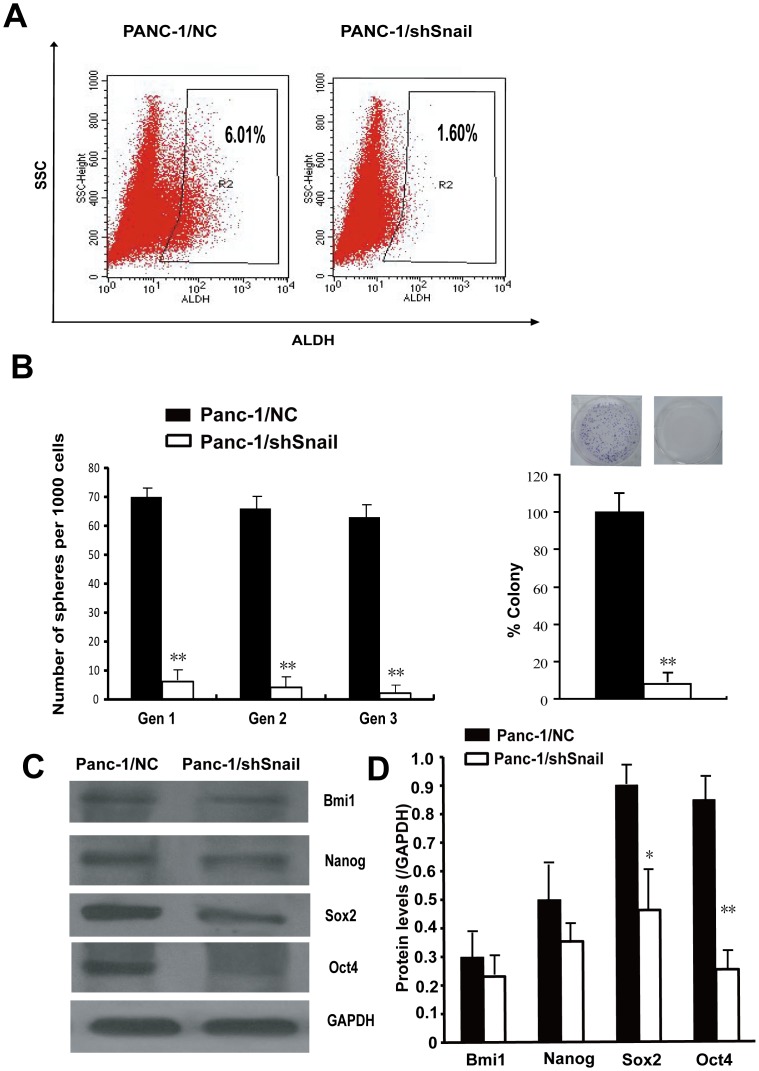
Snail is crucial for properties attributed to cancer stem cells. A. Snail silencing decreases ALDH^high^ population in Panc-1 cells. Dot plots of cells analyzed by flow cytometry for ALDH activity. The values presented are the averages of three independent experiments. B. Snail silencing significantly inhibits the ability of sphere formation with serial passaging and the capability of the clonogenicity in Panc-1 cells. Representative pictures of colony are shown above the column diagram. Similar experiments were repeated three times. ** P<0.01, compared with Panc-1/NC. C. Western blot analysis of cell extracts from Panc-1/NC and Panc-1/shSnail cells for Bmi1, Nanog, Sox2, and Oct4 expression. GAPDH was used as a loading control. D. Quantification of protein levels of Bmi1, Nanog, Sox2, and Oct4 in Panc-1/NC and Panc-1/shSnail cells. * P<0.05, **P<0.01 compared with Panc-1/NC.

As Snail is highly expressed in CSC^high^ compared to CSC^low^ cells, we therefore examined whether Snail could affect the expression of stem cell marker ALDH. Snail silenced Panc-1 cells showed a significant decrease in the ALDH^high^ population (1.60%) in comparison to their control counterparts (6.01%) (Figure 4 A). Next, we used in vitro sphere formation assay to examine whether Snail participates in CSC renewal upon serial passaging. We showed that Snail knockdown not only affected the initial formation of spheres, but also led to an ongoing reduction of sphere numbers in subsequent generations. Colony formation test also showed that silencing of Snail significantly blocked colony growth of Panc-1 cells (Figure 4 B). These experiments implicate that Snail has an important role in the regulation of pancreatic CSC content and is necessary for the self-renewing capacity.

### Snail increases the expression of stem cell associated transcription factors

As Snail expression has some relationship with CSC content, we used Western blotting to determine if Snail expression has roles in changing expression of Bmi1, Nanog, Sox2, and Oct4, which are required for maintaining pluripotency in stem cells. As shown in Figure 4 C and D, lentiviral mediated transfection of Snail shRNA induced a dramatic reduction in the expression of Sox2 and Oct4 (P<0.05 and P<0.01, respectively) in Panc-1 cells, which was consistent with the loss of CSC phenotype, suggesting that the expression of these factors may be important to Snail-induced CSC formation in pancreatic cancer cells.

### Snail enhances pancreatic cancer cell tumorigenicity in vivo

**Figure 5:**
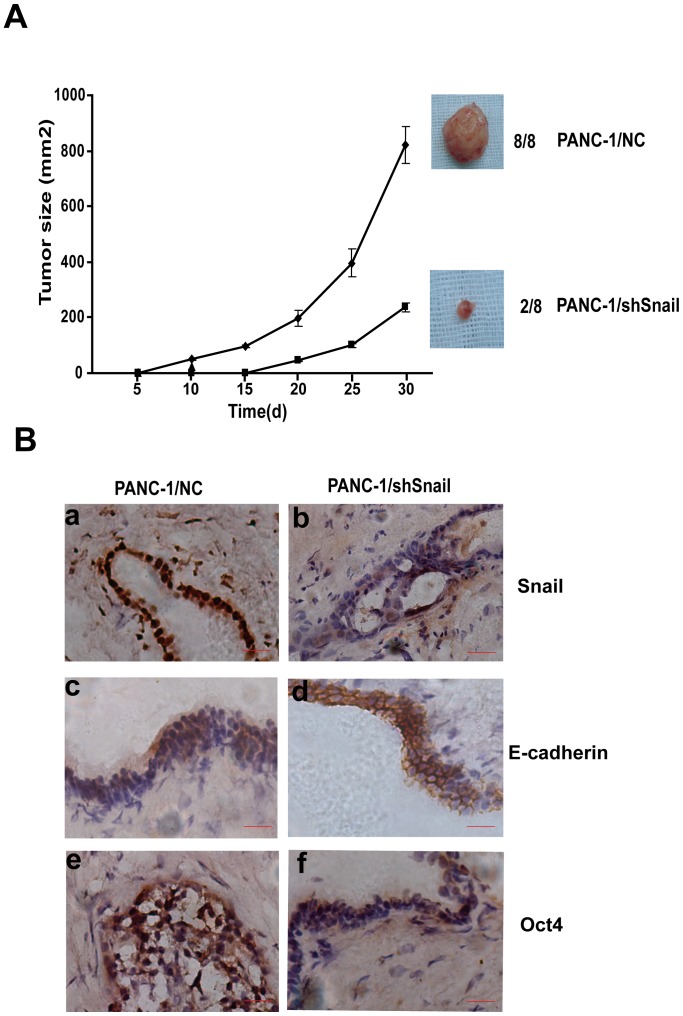
Validation of the role of Snail in tumorigenicity in mouse. A. Graphical representation of growth rates of subcutaneous xenograft tumors using cells of Panc-1/NC and Panc-1/shSnail. 2×10^5^ of each cell was transplanted in eight mice per group. All mice in the Panc-1/NC group, while only 2 mice in the Panc-1/shSnail group had tumor formation. B. Expression of Snail, E-cadherin, and Oct4 in xenograft tumors. Representative examples of Snail, E-cadherin, and Oct4 expression determined by immunohistochemistry. Strongly positive Snail expression is present in the nucleus and cytoplasm of Panc-1/NC tumors (a) but not in Panc-1/shSnail tumors (b). Expression of E-cadherin is weak and heterogeneous in the Panc-1/NC tumors (c), but more intensive in the membrane of Panc-1/shSnail tumors (d). Lower level of Oct4 expression is present in the Panc-1/shSnail tumors (f), as compared to that of Panc-1/NC tumors (e). Scale bars: 10 µm.

Since our in vitro studies suggested that Snail plays a regulatory role in pancreatic cancer cell invasion and CSC formation, we implanted subcutaneously equal numbers of Panc-1/NC and Panc-1/shSnail in the nude mice and measured the resultant tumor growth. When injected 2×10^5^ cells, Panc-1/NC had 100% (8/8) tumor formation while only 2/8 mice injected with Panc-1/shSnail showed pancreatic tumor engraftment. Moreover, a trend to delayed tumor formation and a decrease in tumor sizes were observed in tumors derived from Snail silencing cells compared to those from control cells (Figure 5A). Immunohistochemistry of tumors showed a lighter staining of Snail and Oct4, while a stronger membrane staining of E-cadherin in Panc-1/shSnail tumors, as compared with those of Panc-1/NC tumors (Figure 5B). These data are in accord with our in vitro observations and support the role of Snail in the maintenance of the pancreatic CSC compartment.

## Discussion

Our study demonstrates that Snail which is related to EMT of human pancreatic cancer cells can also regulate expression of stem cell markers and pluripotency maintaining transcription factors, modulating self-renewal capacity and clonogenicity. Together with the tumor implantation study, our results indicate that the activation of Snail is required for the maintenance of cancer stem cell-like traits, which directly impacts tumor initiation, growth in vivo. We have convincingly demonstrated that inhibition of Snail could be considered as a novel strategy to enhance the biological effects of anticancer and chemopreventive agents.

Researchers usually identified CSCs from pancreatic cancer based on the expression of the cell surface antigens like CD44, CD24, epithelial-specific antigen (ESA), and CD133 [17]–[20]. There are different conclusions in separate studies as to which marker best enriches for pancreatic CSCs. However, recent studies have found that cell populations enriched for high ALDH activity alone are sufficient for efficient tumor-initiation with enhanced tumorigenic potential[21]. In our experiment, knockdown of the key EMT inducer Snail significantly decreased the ALDH^high^ cell population. These data indicate that EMT endows tumor cells with stem cell-like properties. Our results are in accordance with the research from Chen et al, which found ALDH^high^ cells in head and neck squamous cancer having EMT shifting and endogenously co-expressed Snail. Furthermore, the knockdown of Snail expression significantly decreased the expression of ALDH, inhibited cancer stem-like properties[22]. These observations were further confirmed by sphere formation in vitro and tumor implantation in vivo, where Snail knockdown led to less and smaller engraftment. These findings are consistent with those of Mani and colleagues [4]who have showed that forced expression of EMT-associated molecules such as Snail and Twist results in cells with a cancer stem cell phenotype. EMT offers an alternative way to generate cancer stem cell properties from differentiated epithelial tumor cells. This hypothesis of dedifferentiation is supported by the recently described generation of pluripotent stem cells from seemingly terminally differentiated somatic cells[23].

It is generally considered that Sox2 and Oct4 are key transcription regulators of embryonic stem cell (ESC) self-renewal and pluripotency[24]–[26]. Accumulating evidence indicates that these transcription factors of ESCs are expressed by CSC-like cells in different types of cancer. Knockdown of these genes could lead to diminished CSC phenotype, reduce the clonogenic and tumorigenic capabilities of cancer cells. [27]–[29]. They are also sufficient to reprogram human somatic cells to pluripotent stem cells that exhibit the essential characteristics of ESCs[23]. In the present study, we found that the expression levels of Sox2 and Oct4 were decreased in Snail-silencing Panc-1 cells compared with the control cells. This suggests that Snail functions as a master switch during regulating the pluripotent potentials of the stem cell. Similar results are found in other researches, in which high expression of Oct4 and Nanog promotes EMT, and is associated with drug resistance, tumor metastasis and poor prognosis in various human malignances.[30], [31]. Our findings, together with the previous studies strongly indicates a molecular link or cross-talk between pluripotency factors and EMT regulatory factors, and thus the activation of these signaling pathways appears to be mechanistically associated with the acquisition of EMT and CSC phenotype of pancreatic cancer cells.

EMT is an embryonic program in which epithelial cells lose their characteristics and gain mesenchymal features. Accumulating evidence suggests that EMT plays an important role during malignant tumor metastasis. It is believed that sustained metastatic growth requires the dissemination of a CSC from the primary tumor followed by its reestablishment in a secondary site. Rhim and colleagues have found that EMT causes epithelially-derived cells to migrate into bloodstream, and seed in liver even before pancreatic tumor formation at the primary site[32]. It is now accepted that aberrant activation of an EMT-stemness program, which are characteristics of cancer cells at the invasive edge of tumor, separates a few of the tumor cells from the primary lesion and exhibits stem cell properties, enables migrating cancer stem cells (MCSCs) to enter blood vessels. The MCSCs are not easily eliminated by conventional therapies due to their drug resistance and quiescence state. Therefore they can seed into distant organs, form micrometastases and finally colonize to macrometastases[33]–[35]. This process is associated with the activation of genes like Snail, which is triggered by environmental factors such as inflammation and hypoxia. Interestingly, Zhang et al demonstrated that hypoxia-stabilized HIF1α promoted EMT through increasing Snail transcription in hepatocellular carcinoma cells[36]. TNFα, a key regulator of the inflammatory response, can also increase the expression of transcription factor Snail and induce EMT in colorectal cancer cells[37].

The highly conserved EMT is frequently associated with the downregulation of E-cadherin and upregulation of vimentin and several transcription factors including Snail, ZEB1 and Slug. Recent researches have identified a link between p53, microRNA miR-34, and Snail in the regulation of cancer cell EMT programs. In the absence of wild-type p53 function, Snail-dependent EMT is activated in cancer cells as a consequence of a decrease in miR-34 levels[38]. Conversely, the transcription factors Snail bound to E-boxes in the miR-34 promoters, thereby repressing miR-34 expression[39]. So, miR-34 and Snail form a double-negative feedback loop to regulate epithelial-mesenchymal transitions. Further studies are needed to clarify whether this feedback loop functions in the EMT-stemness program. Expression of pri-miR-200c is also repressed by Snail. The inhibition of ZEB1 by shSnail in our study may be due to the increased expression of miR-200c, since ZEB1 is a known target of miR-200 c [40].

Based on the current study, Snail is thought to be a critical component of the machinery that maintains CSC compartment. Considering that Snail is just one of EMT regulators, the different contributions of molecular machinery, including the Slug, ZEB1 signaling pathways, might also influence the CSC phenotype. Further analyses would be necessary to clarify the mechanisms underlying the regulation of CSC in pancreatic cancer. Thus, the discovery of molecular knowledge related to CSC characteristics and EMT in pancreatic cancer is becoming an important area of research, and such knowledge is likely to be helpful in the discovery of novel molecular targets and strategies for the prevention of pancreatic cancer by targeting CSCs.

## Supporting Information

Figure S1 **Anti-Snail antibody validation against cell lysates from Panc-1, MIA-PaCa2, BxPC-3, and Capan-1 cell lines.**
(TIF)

Figure S2 **Snail mRNA and protein expression in Panc-1 cells after stable and transient negative control shRNA transfection.** A. Panc-1 cells were transfected by lentivirus-mediated negative control shRNA (NC) or plasmid-mediated negative control shRNA (NC-p). Snail mRNA expression was evaluated by Real-time RT-PCR. B. Protein levels of Snail in stable and transient negative control shRNA-expressing Panc-1 clones.
(TIF)
