# Supplementary figures and images for: Snail Contributes to the Maintenance of Stem Cell-Like Phenotype Cells in Human Pancreatic Cancer
Source: PLoS One. 2014 Jan 29;9(1):e87409. doi: 10.1371/journal.pone.0087409 (PMC3906155; doi:10.1371/journal.pone.0087409)

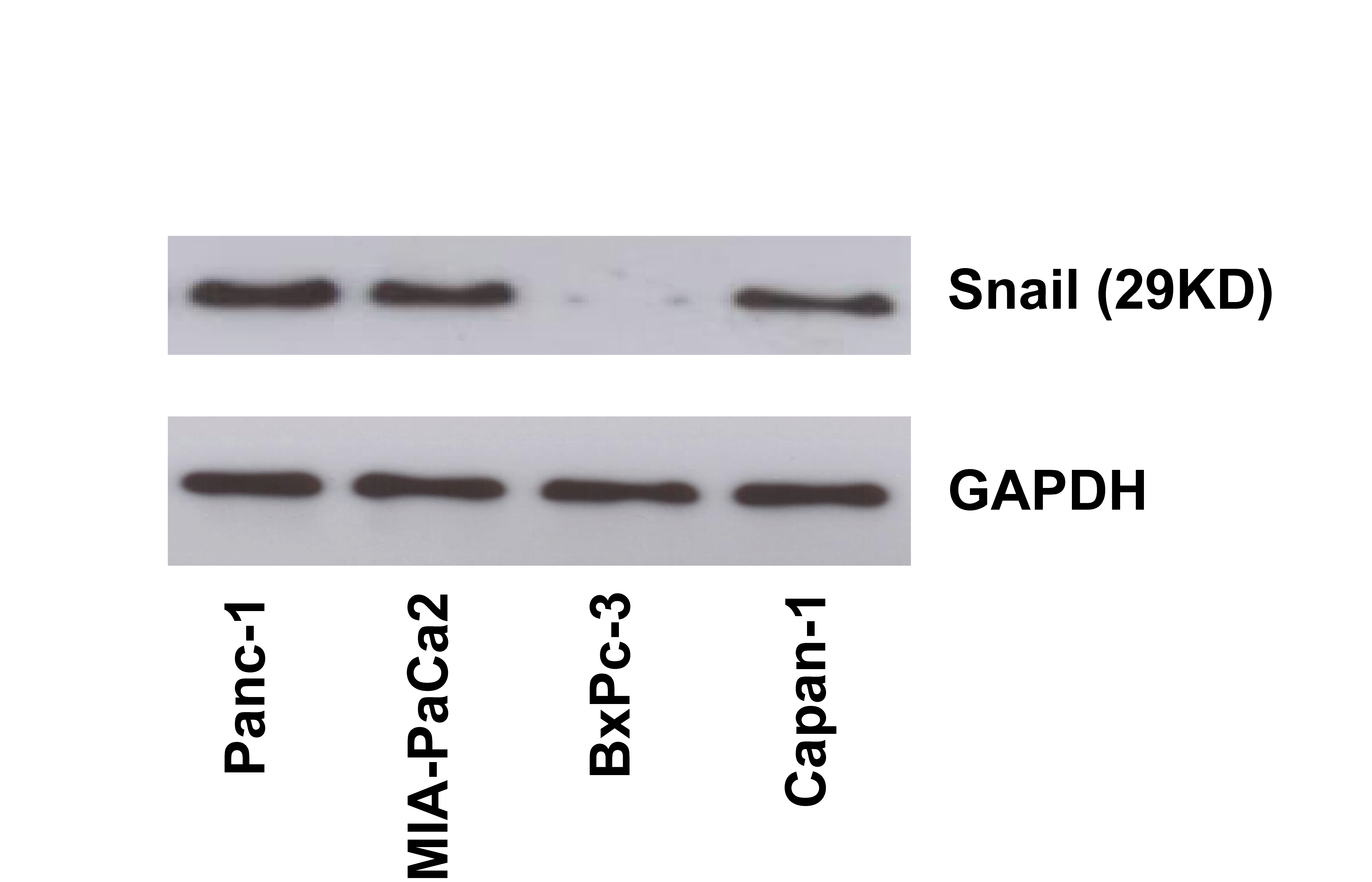

Supplement: Figure S1 — Anti-Snail antibody validation against cell lysates from Panc-1, MIA-PaCa2, BxPC-3, and Capan-1 cell lines. (TIF) [file pone.0087409.s001.tif]

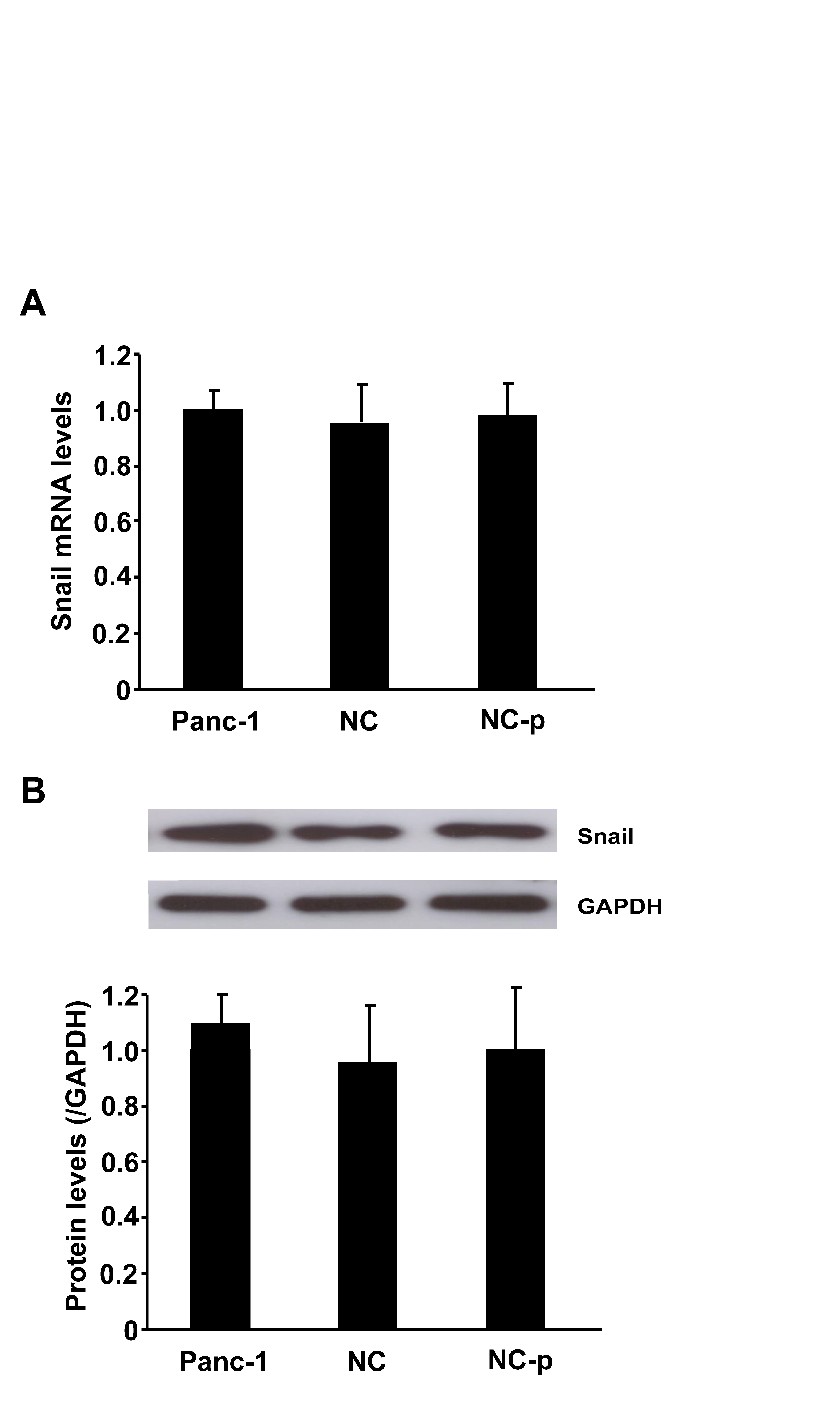

Supplement: Figure S2 — Snail mRNA and protein expression in Panc-1 cells after stable and transient negative control shRNA transfection. A. Panc-1 cells were transfected by lentivirus-mediated negative control shRNA (NC) or plasmid-mediated negative control shRNA (NC-p). Snail mRNA expression was evaluated by Real-time RT-PCR. B. Protein levels of Snail in stable and transient negative control shRNA-expressing Panc-1 clones. (TIF) [file pone.0087409.s002.tif]
